# Factors influencing the implementation of antibiotic de-escalation and impact of this strategy in critically ill patients

**DOI:** 10.1186/cc12819

**Published:** 2013-07-12

**Authors:** Leslie Gonzalez, Aurélie Cravoisy, Damien Barraud, Marie Conrad, Lionel Nace, Jérémie Lemarié, Pierre-Edouard Bollaert, Sébastien Gibot

**Affiliations:** 1 Service de Réanimation Médicale, Hôpital Central, 29 avenue du Maréchal de Lattre de Tassigny, 54035 Nancy Cedex, France

## Abstract

**Introduction:**

A rational use of antibiotics is of paramount importance in order to prevent the emergence of multidrug resistant bacteria that can lead to therapeutic impasse, especially in intensive care units (ICUs). A de-escalation strategy is therefore naturally advocated as part of better antibiotics usage. However, the clinical impact of such a strategy has not been widely studied. We aimed to assess the feasibility and the clinical impact of a de-escalation strategy in a medical ICU and to identify factors associated when de-escalation was possible.

**Methods:**

We performed a retrospective study of patients hospitalized in a medical ICU over a period of six months. Independent factors associated with de-escalation and its clinical impact were assessed.

**Results:**

Two hundred and twenty-nine patients were included in the study. Antibiotics were de-escalated in 117 patients (51%). The appropriateness of initial antibiotic therapy was the only independent factor associated with the performance of de-escalation (OR = 2.9, 95% CI, 1.5-5.7; *P *= 0.002). By contrast, inadequacy of initial antibiotic therapy (OR = 0.1, 0.0 to 0.1, *P *<0.001) and the presence of multidrug resistant bacteria (OR = 0.2, 0.1 to 0.7, *P *= 0.006) prevented from de-escalation. There were no differences in terms of short (ICU) or long-term (at 1 year) mortality rates or any secondary criteria such as ICU length of stay, duration of antibiotic therapy, mechanical ventilation, incidence of ICU-acquired infection, or multi-drug resistant bacteria emergence.

**Conclusions:**

De-escalation appears feasible in most cases without any obvious negative clinical impact in a medical ICU.

## Introduction

The fight against multidrug resistant (MDR) bacteria in health-care facilities is a national priority that involves the whole community and especially intensive care units (ICUs) as they can be considered 'factories' for creating, disseminating, and amplifying resistance to antibiotics [1]. Rational use of antibiotics along with cross-transmission prevention is a crucial part of a strategy aiming at reducing the selection pressure.

Thereby, de-escalation strategy is recommended to prevent the emergence of resistant bacteria. This strategy consists of switching from a broad-spectrum empiric antimicrobial therapy (effective initial treatment) to a narrower spectrum after a systematic reassessment within 72 hours after treatment initiation, depending on the microbiological data obtained [2]. Although de-escalation might be safe and feasible in most patients and during most infections, a surprisingly low number of studies have evaluated this strategy [3] in settings other than ventilator-associated pneumonia [4]. Thus, further information in regard to de-escalation consequences on safety and mortality is clearly needed.

Benefits on the reduction of antibiotic use and shorter duration of therapy are supported by several studies, but the potential consequences of the reduction of antibiotic pressure such as the reduction of MDR bacteria carriage are not precisely detailed. A higher proportion of MDR bacteria carriage when antibiotics were not de-escalated was noticed by Morel and colleagues [3], but other data are scarce.

Several studies highlighted the importance of predefined antibiotic strategies in ICUs on the basis of patient characteristics, severity and location of infection, and collaboration with microbiologists and infectious disease specialists [5]. Despite the existence of such predefined strategies, de-escalation rate may probably be largely improved. To this end, the determination of factors influencing the implementation of de-escalation is important in order to increase its use.

Here, we retrospectively assessed the clinical and microbial impact of the use of a de-escalation strategy in a medical ICU. We also tried to elucidate factors influencing the implementation of de-escalation.

## Materials and methods

### Study design and patients

This single-center retrospective study included patients hospitalized in the 14-bed medical intensive care department of the Nancy University Central Hospital in France. The CPP-Est III has approved this study, and owing to the retrospective nature of the study, patient consent was waived. Charts pertaining to 365 consecutive adult patients (more than 18 years old) admitted within a 6-month period were retrospectively reviewed. Patients who never received antibiotics or benefited from an antibioprophylaxis, those in whom infection was not suspected, or those who spent less than 72 hours in the ICU were excluded.

### Data extraction

We recorded the following: demographic characteristics (age and gender), underlying diseases such as immunosuppression (corticosteroids, long-term immunosuppressive administration, HIV infection, and splenectomy), diabetes, chronic respiratory insufficiency, chronic renal failure, chronic heart failure, hypertension, chronic alcohol abuse, smoking, nutritional status, obesity, and allergy to penicillin. Severity of underlying disease was evaluated by McCabe score [6]. The severity of illness on admission was assessed by using the Simplified Acute Physiology Score II (SAPS II) [7] and during hospitalization by using the Sequential Organ Failure Assessment (SOFA) score [8]. The reason for admission was also recorded.

For all patients, data, including body temperature, corticosteroid therapy, SOFA score, and usual biological parameters, were collected daily until day 8 and then at days 10, 14, 21, and 28. Use of mechanical ventilation and vasopressors, ICU length of stay, and short-term (ICU, 28 days, and hospital) and long-term (12 months) mortality rates were also recorded.

The site of infection, suspected or documented, was assessed according to standard criteria [9]: pneumonia was established by the presence of new infiltrates on chest radiograph, body temperature of more than 38°C or white blood cell count of less than 4,000/mm^3 ^(or more than 12,000/mm^3^) or both, and at least one of the following criteria: new onset of bronchial purulent sputum, alteration of arterial oxygenation, and evocative pulmonary auscultation. Urinary tract infection was defined by the presence of microorganism at the concentration of at least 10^5 ^colony-forming units per milliliter (CFU/mL) with symptoms or urinary catheter or both. Abdominal infection was defined by the involvement of organs of the abdominal cavity, including stomach, spleen, pancreas, small intestine, large intestine, and gallbladder. We also collected gynecologic (uterus and ovaries), skin, and soft tissues and neurologic, bone, ear-nose-throat, blood, and mediastinal infections data. Bacterial infection was confirmed when microorganisms grew more than 10^5 ^CFU/mL in urine, more than 10^4 ^CFU/mL (for bronchoalveolar lavage), more than 10^5 ^CFU/mL (tracheal aspiration), more than 10^6 ^CFU/mL (expectoration) in lung, or when present in culture of otherwise-sterile fluids or tissues (cerebrospinal, pleural, peritoneal, gynecologic, bone, and blood) or devices (quantitative diagnosis), or in case of positivity of urinary antigens of *Streptococcus pneumoniae *or *Legionella pneumophilia*.

A search for MDR bacteria carriage was performed (by nasal and rectal swabs) at admission and then once a week. MDR bacteria were defined as the following: methicillin-resistant *Staphylococcus aureus *and coagulase-negative staphylococci; Enterobacteria producing an extended-spectrum beta-lactamase or producing a cephalosporinase; and ticarcillin-resistant *Acinetobacter baumanii *and piperacillin-tazobactam-resistant *Pseudomonas aeruginosa *[10]. Finally, incidence of naturally resistant bacteria (*Enterococcus faecium *and *E. faecalis, Enterobacter cloacae, Clostridium difficile, Corynebacterium, Serratia marcescens, Providencia*, and *Stenotrophomonas maltophilia*) was also recorded [11].

### Antibiotics

Empiric antibiotic therapy is not protocolized in our unit: it was prescribed by the physician in charge of the patient on the basis of patient medical history, characteristics, severity, suspected site of infection, and hospital ecology. Every antibiotic prescription was then discussed daily during a morning meeting with the medical staff. Clinical pharmacists reviewed all drug prescriptions. We recorded type of antibiotics, delay of administration, and adequacy with microbiological data.

De-escalation therapy was defined as the reduction in the number of antibiotics or spectrum narrowing before the fifth day of antibiotherapy. 'Antibiotic discontinuation' was defined as all antibiotic withdrawal within 3 days. De-escalation was not protocolized and was performed by the physician in charge of the patient in accordance with the patient's clinical evolution, and bacterial identification and antibiotic susceptibility data were systematically checked every day; this assessment began within 24 hours after admission. Spectrum narrowing was carried out on the basis of susceptibility data; reduction in the number of antibiotics was usually achieved in the case of spectrum narrowing or when switching from beta-lactam + macrolide or fluoroquinolone to beta-lactam alone in the case of community-acquired pneumonia, even in the absence of microbial documentation (except in the case of *L. pneumophila *suspicion). When no obvious infectious site was evidenced, early antibiotic discontinuation was performed within the first 3 days provided that the clinical evolution was favorable or that an alternate diagnosis was found. Escalation was defined as the addition of a new antibiotic or a switch for a broader-spectrum agent. Every antibiotic change was discussed on the following day (except on weekends) with the rest of the medical staff during a morning meeting. Once a week, this meeting also included a microbiologist and infectious disease specialists.

Antimicrobial therapy was considered appropriate when at least one of the antibiotics had an *in vitro *activity against the identified microorganism. The pharmacodynamics of prescribed antibiotics was not analyzed, as data were not available. The definition of de-escalation strategy allowed us to create two groups for subsequent analysis of associated factors: 'de-escalation' group (D) and 'no de-escalation' group (ND).

### Statistical analysis

Descriptive results of continuous variables were expressed as mean ± standard deviation or as median (interquartile range), depending on the normality of their distribution. Variables were tested for their association with de-escalation by using Pearson's chi-squared test for categorical data and Mann-Whitney *U *test for numerical data. A multiple stepwise logistic regression model was established with any covariate with univariate significance of a *P *value of less than 0.10 eligible for inclusion in the model. The model was then further calibrated through Hosmer-Lemeshow testing. To elucidate the effect of de-escalation on mortality, survival curves were constructed and Kaplan-Meier testing was used.

## Results

### Population description

**Figure 1:**
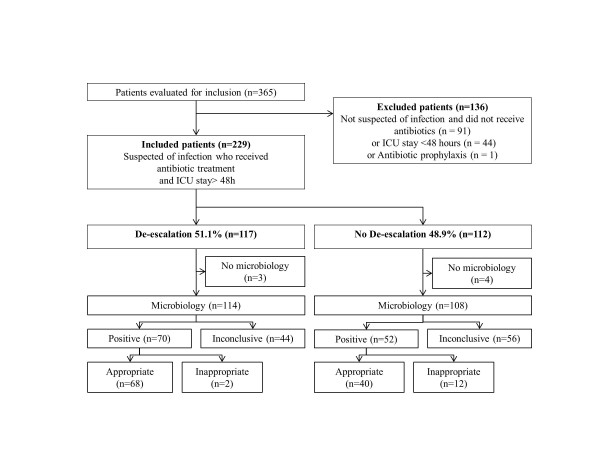
**Flow chart of study selection process and classification according microbiological data and therapeutic strategy**. ICU, intensive care unit.

We reviewed the charts of 365 patients: 136 were excluded because they were not suspected of infection and did not receive antibiotics or because they spent less than 72 hours in the ICU (Figure 1). Two hundred twenty-nine patients (mean age of 59.5 ± 17 years) were thus included in this study, and only the first suspected infectious episode was analyzed. Demographic and infection-related data are presented in Table 1. Patients were more often male (57.2%) and had a mean McCabe score of 1.1 ± 0.8, a SAPS II of 51 ± 19, and a SOFA score of 7.5 ± 4.6 at admission. The main reasons for admission were septic shock (35.8%), coma (23.1%), and acute respiratory failure (20.5%). Suspected infection was community-acquired in 72.5% (*n *= 166) of cases.

**Table 1:** Characteristics according to the therapeutic strategy

Characteristics	All patients (*n *= 229)	De-escalation (*n *= 117)	No de-escalation (*n *= 112)	*P *value
Age, years	59.5 ± 17.0	61.2 ± 17.3	57.7 ± 16.7	0.11

Sex, number (percentage)				0.04
Male	131 (57.2)	62 (53)	69 (61.6)	
Female	98 (42.8)	55 (47)	43 (38.4)	

McCabe score	1.1 ± 0.8	1.1 ± 0.8	1.1 ± 0.8	0.78

History of immunosuppression, number (percentage)	22 (9.6)	11 (9.4)	11 (9.8)	0.62

SAPS II	51 ± 19	51 ± 19	50 ± 18	0.77

SOFA score	7.5 ± 4.6	7.8 ± 4.6	7.2 ± 4.5	0.32

Recent hospitalization or home care, number (percentage)	36 (15.7)	20 (17.1)	16 (14.3)	0.31

Reasons for admission, number (percentage)				
Acute respiratory failure	47 (20.5)	28 (23.9)	19 (16.9)	0.03
Coma	53 (23.1)	15 (12.8)	38 (33.9)	<0.001
Septic shock	82 (35.8)	52 (44.4)	30 (26.8)	<0.001
Miscellaneous	47 (20.5)	22 (18.8)	25 (22.3)	0.29

Previous antiotherapy within 24 hours, number (percentage)	50 (21.8)	32 (27.3)	18 (16.1)	<0.001

Mechanical ventilation, number (percentage)	168 (73.4)	86 (73.5)	82 (73.2)	0.73

Vasopressors, number (percentage)	111 (48.4)	68 (58.1)	43 (38.4)	<0.001

Body temperature, °C	38.3 ± 1.9	39.7 ± 1.7	36.9 ± 1.9	<0.001

Leucocytes count, g/L	15.7 ± 12.2	17.1 ± 14.6	14.4 ± 8.9	0.09

Procalcitonin, ng/mL	18.9 ± 46.0	13.7 ± 28.9	23.8 ± 58.2	0.09

Suspicion of community-acquired infection, number (percentage)	166 (72.5)	90 (77.9)	76 (67.8)	0.02

Suspected infection site, number (percentage)				
Lung	157 (68.5)	76 (64.9)	81 (72.3)	0.06
Urinary tract	18 (7.9)	12 (10.2)	6 (5.3)	0.01
Abdomen	24 (10.5)	14 (11.9)	10 (8.9)	0.20
Soft and skin tissues	11 (4.8)	5 (4.2)	6 (5.3)	0.47
Other sites	19 (8.3)	10 (8.6)	9 (8.0)	0.60

Infection finally ruled out, number (percentage)	37 (16.2)	15 (12.8)	22 (19.6)	0.05

Infection documented, number (percentage)	122 (53.3)	70 (59.8)	52 (46.4)	0.002

MDR, number (percentage)	21 (17.2)	8 (11.4)	13 (25)	<0.001

Bacteremia, number (percentage)	42 (18.3)	24 (20.5)	18 (16.1)	0.15

Empiric antibiotherapy, number (percentage)				
Appropriate	108 (47.2)	68 (58.1)	40 (35.7)	<0.001
Not appropriate	14 (6.1)	2 (1.7)	12 (10.7)	<0.001
Unknown	107 (46.7)	47 (40.2)	60 (53.6)	0.003

Duration of mechanical ventilation	7.7 1 ± 2.1	8.3 ± 11.7	7.2 ± 12.6	0.49

Duration of catecholamine administration	4.9 ± 4.8	5.4 ± 5.8	4.2 ± 4.8	0.16

Duration of antibiotic administration	7.7 ± 8.0	7.9 ± 6.4	7.5 ± 9.4	0.70

ICU-acquired infections, number (percentage)	9 (3.9)	3 (2.5)	6 (5.3)	0.15

ICU length of stay	11.5 ± 14.4	12.9 ± 15.6	10.0 ± 12.9	0.12

Mortality, number (percentage)				
ICU	41 (17.9)	20 (17.1)	21 (18.7)	0.50
Hospital	60 (26.2)	30 (25.6)	30 (26.8)	0.58

*P *values are comparisons between De-escalation and No De-escalation groups. SAPS II, Simplified Acute Physiologic Score II; ICU, intensive care unit; MDR, multidrug resistant; SOFA, Sepsis-related Organ failure Assessment.

Infectious sites were lung (55%) (*n *= 126), urinary tract (9.6%), or abdomen (9.6%). Infection was confirmed in 83.8% (*n *= 192) of patients. Fifty (21.8%) patients already received antibiotics at the time of ICU admission (for more than 24 hours), but antibiotics were proven appropriate in only 38% (*n *= 19).

Microbiological examinations yielded to a bacteriological diagnosis in 53.3% (*n *= 122) of cases. Identification was achieved mainly on alveolar lavage fluid samples in 54.1% (*n *= 66) of these cases or on urinary samples in 15.6% (*n *= 19). Bacteremia was present in 18.3% (*n *= 42) of patients. Enterobacteria were the most often identified pathogens (38.5%, *n *= 47), followed by *Staphylococcus sp*. (28.7%, *n *= 35), and *Streptococcus pneumonia *(19.7%, *n *= 24). In 34 cases (27.9%), several pathogens were identified. The most frequently prescribed empirical antibiotics were quinolones (48.9%, *n *= 112), group A penicillins (43.2%, *n *= 99), third-generation cephalosporins (33.2%, *n *= 76), carboxy and ureido-penicillins (17%, *n *= 39), and linezolid (12.2%, *n *= 28) (Table 2). The initial empirical antibiotic treatment was effective in 45.8% of cases, inappropriate in 6.1%, and of uncertain appropriateness (because of a lack of microbial findings) in 46.7%.

**Table 2:** Initial antibiotherapy

Initial antibiotics, number (percentage)	All patients (*n *= 229)	De-escalation (*n *= 117)	No de-escalation (*n *= 112)	*P *value
Group A penicillins	99 (43.2)	30 (25.6)	69 (61.6)	<0.001

Carbapenems	10 (4.4)	8 (6.8)	2 (1.8)	<0.001

Carboxy and ureido-penicillins	39 (17.0)	20 (17.1)	19 (17.0)	0.8

Fluoroquinolones	112 48.9)	78 (66.7)	34 (30.4)	<0.001

Glycopeptides, linezolid	45 (19.6)	28 (23.9)	17 (15.2)	0.01

Cephalosporins	76 (33.2)	56 (47.9)	20 (17.9)	<0.001

Macrolides	15 (6.6)	13 (11.1)	2 (1.8)	<0.001

Aminoglycosides	17 (7.4)	8 (6.8)	9 (8.0)	0.7

Nitroimidazole	19 (8.3)	12 (10.3)	7 (6.3)	0.05

Others Number of antibiotics	11 (4.8)	8 (6.8)	3 (2.7)	0.003

1	79 (34.5)	11 (9.4)	68 (60.7)	<0.001

2	93 (40.6)	73 (62.4)	20 (17.9)	<0.001

3	50 (21.8)	28 (23.9)	22 (19.6)	0.2

4	5 (2.2)	4 (3.4)	1 (0.9)	<0.001

5	2 (0.9)	1 (0.9)	1 (0.9)	0.9

### De-escalation

De-escalation was performed in 51.1% (*n *= 117) of included patients 3.8 ± 1.5 days after the introduction of empirical antibiotic therapy. In these patients, a bacterial documentation was obtained in 57.4% (*n *= 70). The number of antibiotics was decreased for 110 (94%) patients, the spectrum was reduced for 7 (6%) patients, and antibiotics were completely stopped for 11 (9.4%) patients ('antibiotic discontinuation'). The de-escalated antibiotics were mainly quinolones (57.7%), linezolid (21%), and third-generation cephalosporins (10.3%). Polymicrobial isolates were identified in 34 patients, and de-escalation was achieved in 19 patients by reducing the number of antibiotics in 16 patients and narrowing the spectrum in three.

Among the 117 'de-escalated' patients, only three patients did not have microbiological sampling and antibiotics were withdrawn (two end-of-life decisions and one non-infectious diagnosis). Among the remaining 114 patients, microbiological identification was achieved in 70.

Next, we sought to study factors associated with the implementation of de-escalation (Table 1). In univariate analysis, de-escalated patients, as compared with non-de-escalated, were more often females, had high body temperature, and more often presented with septic shock, urinary infection, or community-acquired infection. Initial antibiotic therapy was more often appropriate, and the identification of a responsible microorganism was more frequent. Finally, differences in regard to the initial antibiotics used also existed (Table 2).

Among these factors, only the appropriateness of initial antibiotic therapy - odds ratio (OR) = 2.9, 95% confidence interval (CI) = 1.5 to 5.7, *P *= 0.002 - was associated with the performance of de-escalation in multivariate analysis (Table 3). By contrast, narrow-spectrum initial antibiotic therapy (OR = 0.1, 95% CI = 0.0 to 0.1; *P *<0.001) and infection with an MDR bacteria (OR = 0.2, 95% CI = 0.1 to 0.7, *P *= 0.006) prevented de-escalation.

**Table 3:** Multivariate logistic regression analysis to assess factors associated with the realization of de-escalation

Variable	Coefficient	Standard error	Chi-squared	*P *value	Odds ratio (95% CI)
Sex	−0.32	0.34	0.9	0.33	0.7 (0.4-1.4)

Coma	−0.21	0.47	0.2	0.66	0.8 (0.3-2.0)

Urinary tract infection	0.24	0.61	0.2	0.68	1.3 (0.4-4.2)

Previous antibiotherapy	0.63	0.41	2.3	0.13	1.9 (0.8-4.2)

Appropriate initial antibiotherapy	1.08	0.34	9.9	0.002	2.9 (1.5-5.7)

Narrow-spectrum antibiotic	−4.51	1.04	18.6	<0.001	0.1 (0.0-0.1)

MDR bacterial infection	−1.41	0.52	7.4	0.006	0.2 (0.1-0.7)

CI, confidence interval; MDR, multidrug resistant.

### No de-escalation

In 112 cases, de-escalation was not performed, and antibiotic escalation (broadening of the spectrum or adding antibiotics) was even performed in 18 patients. Among these 112 patients, a microbiologic identification was obtained in 52: in 23 of them, antibiotics were appropriate with a narrow spectrum; in 12, antibiotics were inadequate (a resistant organism was present in 11). All in all, de-escalation should have been possible in 17 patients.

### Outcome criteria

**Figure 2:**
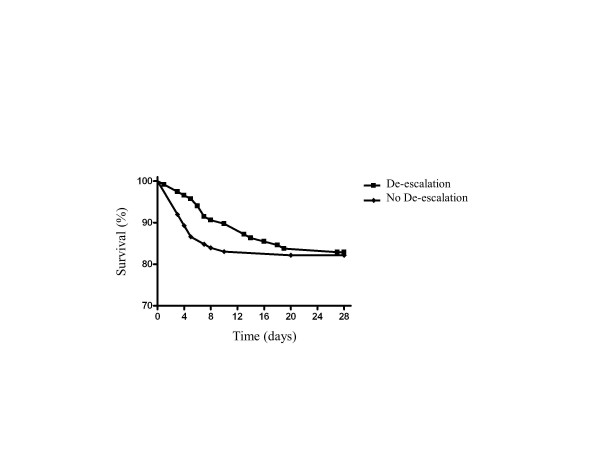
**Kaplan-Meier estimate of survival according to therapeutic strategy**.

Forty-one patients (17.9%) died in the ICU, and 60 died during their hospital stay (26.2%). Mortality rates between the two groups of patients were similar: 17.1% (de-escalation) versus 18.7% (no de-escalation) in the ICU and 25.6% versus 26.8% in the hospital (Table 1). Day-28 Kaplan-Meier estimates of survival also showed no differences between de-escalated and non-de-escalated patients (Figure 2). Mortality was also identical between groups at up to 12 months (26.8% versus 26.5% de-escalation versus no de-escalation). De-escalation was performed in 44% of culture-negative patients with a lower ICU mortality rate (9.1% versus 23.2%, *P *= 0.03) but with no differences thereafter (hospital mortality: 22.7% versus 30.4%, *P *= 0.32). De-escalation did not influence ICU length of stay (12.9 ± 15.6 versus 10 ± 12.9 days) or the duration of mechanical ventilation (8.3 ± 11.7 versus 7.2 ± 12.6 days), vasopressors (3.1 ± 4.9 versus 2.2 ± 4.8 days), or antibiotics (7.9 ± 6.5 versus 7.5 ± 9.4 days). The incidence of ICU-acquired infections was also not affected (2.5% versus 5.3%).

Microbiological data allowed the analysis of carriage and infections due to MDR bacteria. We found no significant difference in ICU-acquired infections due to MDR bacteria between the two groups (de-escalation *n *= 2 versus no de-escalation *n *= 0). There was also no difference in regard to MDR bacteria carriage at the last screening (15.3%, *n *= 18 versus 10.7%, *n *= 12; *P *= 0.1).

## Discussion

In this retrospective single-center study on critically ill patients, antibiotics were de-escalated in 117 (51%) patients. This finding is similar to what was observed by Morel and colleagues [3]: in their retrospective study of 116 patients, the de-escalation rate was 45%. Studies focusing on specific subgroups of patients gave various results. The de-escalation rate ranged from 6% to 74% in patients with ventilator-associated pneumonia [12,13] and was 43% in patients with severe sepsis or septic shock [14].

Although the rate of de-escalation we observed appears acceptable in light of the literature, could it have been improved? Among non-de-escalated patients, an initial broad-spectrum antibiotherapy could have been stepped down in 17: thus, there is clear room for improving the rate of de-escalation. In univariate analysis, reasons for no de-escalation were (a) inadequate initial antibiotics (10.7%), (b) lack of microbiological documentation (50%), (c) initial appropriate antibiotic therapy that could not be de-escalated (narrow-spectrum) (20.5%), and (d) clinical worsening despite appropriate antibiotic therapy (7.1%). Eighteen escalations (7.9%) were necessary, and this rate is comparable to those of the study by Morel and colleagues (6.6%) [3] and the study by Leone and colleagues (6%) [13]. Among these 18 escalated patients, hospital mortality was not higher (22.2%, *n *= 4).

In multivariate analysis, when factors associated with de-escalation were looked at, only the appropriateness of initial antibiotherapy was independently associated with its implementation. Unsurprisingly, initial use of narrow-spectrum antibiotics and the presence of MDR bacteria precluded the performance of de-escalation. As initial antibiotics are often started before ICU admission, these factors seem hard to modify.

In this study, the use of fluoroquinolones was frequent (48.9%) because of a high incidence of pulmonary infections (68.5%). Therefore, factors associated with de-escalation may be different in ICUs dealing with a different case mix. Concerns about the safety of such a strategy are natural, especially in patients without any microbial documentation. Here, we observed that de-escalation occurred in 44% of culture-negative patients (Figure 1) without any negative impact on mortality. Therefore, such a strategy may be safe even in the absence of a clear microbial documentation.

The same holds true for the sickest patients: 82 (35.8%) were admitted for septic shock, 52 (63.4%) of whom benefited from de-escalation with no impact on mortality in the ICU (D: *n *= 11, 21.2% versus ND: *n *= 9, 30%; *P *= 0.13) or in the hospital (D: *n *= 17, 32.7% versus ND: *n *= 43.3%; *P *= 0.16). These findings, in terms of feasibility in patients with septic shock, are in line with those of Heenen and colleagues [14]. However, this has to be confirmed in a larger population. Moreover, very few patients had hematological diseases (*n *= 5) or were solid-organ transplant recipients (*n *= 2): the safety of de-escalation in these specific populations also has to be confirmed.

In this study, we did not find any difference in terms of mortality between de-escalation and no de-escalation groups, even when analyzed at up to 1 year, confirming the hypothesis that de-escalation is safe and feasible. These results are also in agreement with those of Morel and colleagues [3]. In some subgroups of patients, de-escalation may even be beneficial. For example, Joung and colleagues [15] observed a decreased mortality rate in de-escalated patients with ICU-acquired pneumonia.

In regard to secondary outcomes such as ICU length of stay and duration of mechanical ventilation or antibiotics use, we were also unable to observe any differences between groups, even if unfortunately we were not able to perform cost analyses.

De-escalation is believed to prevent the emergence of MDR bacteria, although evidence remains scarce in the literature. In the study by Morel and colleagues [3], MDR bacteria acquisition rate was not reduced in de-escalated patients. Again, our findings support this view: MDR ICU-acquired infections were very rare with no influence of de-escalation (*n *= 2). Finally, de-escalation had no impact on MDR bacteria carriage (15.3% in de-escalated patients versus 10.7% in non-de-escalated patients; *P *= 0.1). These results also have to be tempered given the limited number of patients, the single-center design, the relatively short duration of antibiotic therapy, and the lack of antibiotic exposure quantification. Moreover, Kim and colleagues [16] recently reported an increase of MDR bacteria carriage in de-escalated patients with hospital-acquired pneumonia (adjusted hazard ratio 3.84, 95% CI 1.06 to 13.91).

## Conclusions

We demonstrated that, among ICU patients, a strategy of de-escalation in a context of global management with microbiologists, infectious disease specialists, and clinical pharmacists was possible in most cases, including septic shock, and did not influence short- and long-term prognosis or acquisition of MDR bacteria.

## Key messages

• De-escalation is possible in most patients in the intensive care unit.

• The appropriateness of initial antibiotic therapy was the only independent factor associated with its implementation.

• De-escalation is feasible even in case of septic shock.

• De-escalation does not influence short- or long-term outcome.

## Abbreviations

CFU: colony-forming units; CI: confidence interval; D: de-escalation; ICU: intensive care unit; MDR: multidrug resistant; ND: no de-escalation; OR: odds ratio; SAPS II: Simplified Acute Physiology Score II; SOFA: Sequential Organ Failure Assessment.

## Competing interests

The authors declare that they have no competing interests.

## Authors' contributions

SG helped to design the study, to perform acquisition of the data, to perform analysis and interpretation of data, and to draft the manuscript and performed the statistical analysis. LG helped to design the study, to perform acquisition of the data, to perform analysis and interpretation of data, and to draft the manuscript. PEB helped to perform analysis and interpretation of data and critical revision of the manuscript. AC, DB, MC, LN, and JL helped to perform the interpretation of data and critical revision of the manuscript. All authors read and approved the final manuscript.
